# A Review of the Role of Natural Products as Treatment Approaches for Xerostomia

**DOI:** 10.3390/ph16081136

**Published:** 2023-08-10

**Authors:** Konstantinos N. Kontogiannopoulos, Afroditi Kapourani, Ioannis Gkougkourelas, Maria-Emmanouela Anagnostaki, Lazaros Tsalikis, Andreana N. Assimopoulou, Panagiotis Barmpalexis

**Affiliations:** 1 Department of Pharmaceutical Technology, School of Pharmacy, Aristotle University of Thessaloniki, 54124 Thessaloniki, Greece; akapourag@pharm.auth.gr (A.K.); igougourelas@yahoo.com (I.G.); miretserk@gmail.com (M.-E.A.); pbarmp@pharm.auth.gr (P.B.); 2 Department of Preventive Dentistry, Periodontology and Implant Biology, School of Dentistry, Aristotle University of Thessaloniki, 54124 Thessaloniki, Greece; tsalikis@dent.auth.gr; 3 Laboratory of Organic Chemistry, School of Chemical Engineering, Aristotle University of Thessaloniki, 54124 Thessaloniki, Greece; adreana@cheng.auth.gr; 4 Natural Products Research Centre of Excellence-AUTH (NatPro-AUTH), Center for Interdisciplinary Research and Innovation (CIRI-AUTH), 57001 Thessaloniki, Greece

**Keywords:** xerostomia, dry mouth, natural products, artificial saliva, salivary substitutes

## Abstract

Xerostomia, commonly known as dry mouth, is a widespread oral health malfunction characterized by decreased salivary flow. This condition results in discomfort, impaired speech and mastication, dysphagia, heightened susceptibility to oral infections, and ultimately, a diminished oral health-related quality of life. The etiology of xerostomia is multifaceted, with primary causes encompassing the use of xerostomic medications, radiation therapy to the head and neck, and systemic diseases such as Sjögren’s syndrome. Consequently, there is a growing interest in devising management strategies to address this oral health issue, which presents significant challenges due to the intricate nature of saliva. Historically, natural products have served medicinal purposes, and in contemporary pharmaceutical research and development, they continue to play a crucial role, including the treatment of xerostomia. In this context, the present review aims to provide an overview of the current state of knowledge regarding natural compounds and extracts for xerostomia treatment, paving the way for developing novel therapeutic strategies for this common oral health issue.

## 1. General

The term “xerostomia”, originating from the Greek words “xeros” (dry) and “stoma” (mouth), refers to the subjective sensation of oral dryness experienced by patients. However, for quantifiable changes in reduced saliva production, “salivary gland hypofunction” is more accurate, while “salivary gland dysfunction” denotes broader alterations in the physiological salivary gland function [1]. Xerostomia impacts millions of individuals globally, with the literature indicating that xerostomia predominantly affects menopausal women and those over 65 years [2].

Evidence from the literature has demonstrated the therapeutic potential of various herbal extracts in addressing the xerostomia [2,3]. However, the multiple constituents within these extracts present a significant challenge in elucidating the molecular mechanisms and modes of action underlying their therapeutic effects. Researchers are concentrating on isolating individual active therapeutic constituents from herbal extracts to circumvent this issue. Nevertheless, the therapeutic potential and mechanisms of action of these isolated components, derived from plant extracts, in the treatment of xerostomia remain to be fully elucidated [2].

### 1.1. Xerostomia

Xerostomia, alternatively referred to as dry mouth, is characterized by an individual’s subjective perception of oral dryness, often resulting from diminished salivary secretion (hyposalivation) [4]. An individual’s average salivary flow rate is about 0.5–1.5 L daily. Still, this flow rate can be significantly reduced in cases of xerostomia, leading to a range of symptoms and complications [5]. The emergence of xerostomia can be attributed to an array of systemic or localized causative factors [6,7], such as extensive medication usage, head and neck radiation therapy, and systemic pathologies, including diabetes mellitus, sarcoidosis, systemic lupus erythematosus, and the autoimmune disorder known as Sjögren’s syndrome [8,9,10,11]. Upon scrutinizing the existing literature, it is evident that the prevalence of xerostomia in the general population exhibits considerable variation, with reported incidences among adults spanning from 5.5% to 46% [12,13].

Patients suffering from xerostomia often report symptoms that considerably impair their overall health and quality of life, affecting them socially and emotionally. Discomfort associated with oral dryness is the initial and most commonly cited symptom among individuals affected by xerostomia [14,15,16]. Research has provided evidence indicating a notable rise in the occurrence of oral health issues among these patients, including dental decay and fungal infections in the oral cavity, such as candidiasis, oral malodor, glossopyrosis, and periodontitis [17,18,19]. Additionally, dysphagia and dysgeusia have been identified as potential clinical manifestations [20,21].

The etiology of clinical manifestations dictates the necessary therapeutic approach, which frequently demands a multidisciplinary strategy due to its multifactorial origins. General management primarily focuses on palliative care to alleviate symptoms and prevent oral complications. Current therapeutic approaches for xerostomia comprise the use of salivary substitutes, particularly in instances where total damage to the salivary glands has occurred, and the implementation of salivary stimulants in situations where the salivary glands retain some degree of operational capability. Salivary stimulants include masticatory substances like chewing gum for mechanical incitement, malic and ascorbic acids, and parasympathomimetic pharmaceuticals for pharmacological incitement [22,23].

#### Innervation of the Salivary Glands

Salivary glands receive dual innervation from the parasympathetic and sympathetic nervous systems. The parasympathetic system primarily drives serous salivary production and ion secretion, while the sympathetic system enhances protein secretion and modulates glandular blood flow along with local inflammatory and immune mediators. The parotid gland obtains its innervation through the glossopharyngeal nerve (CN IX), with the cell bodies of the parasympathetic fibers located in the otic ganglion. Postganglionic fibers from this ganglion join the auriculotemporal nerve to provide innervation to the parotid gland.

Meanwhile, the submandibular ganglion, positioned within the submandibular gland (SMG) and near the lingual nerve, hosts the cell bodies of parasympathetic fibers that supply the SMG and sublingual gland (SLG), including their myoepithelial cells. Preganglionic parasympathetic fibers for these glands originate from the superior salivatory nucleus in the pons and join the facial nerve (CN VII). These fibers traverse the chorda tympani, exit the skull via the petrotympanic fissure, and combine with the lingual nerve. They run adjacent to Wharton’s duct and synapse in the submandibular ganglion.

Conversely, the sympathetic fibers’ cell bodies are located in the superior cervical ganglion in the neck. These postganglionic fibers innervate the glands alongside blood vessels branching from the carotid plexus. The preganglionic fibers originate in the thoracic ganglion and ascend through the spinal cord. Unlike the major glands, the minor salivary glands’ secretions are not regulated by neuronal control. They consistently secrete small saliva amounts, ensuring continual lubrication of oral surfaces, even when the major glands are in a resting state, such as during the night [24].

### 1.2. Natural Products Used in Pharmaceuticals

Natural products have been used for medicinal purposes for centuries, and today, they play an essential role in pharmaceutical research and development. Natural products are chemical compounds derived from plants, animals, marine organisms, and microorganisms. They have been found to possess a wide range of biological activities, including antibacterial, antifungal, anticancer, and anti-inflammatory properties [25,26]. The use of natural products in pharmaceuticals has several advantages over synthetic drugs. Natural products are often more readily available and less expensive than synthetic compounds, being rendered an attractive option for drug development. In addition, natural products have a long history of use in traditional medicine, providing a wealth of knowledge and experience for researchers to draw upon. Furthermore, natural products have a high degree of structural diversity, making them an excellent source of novel chemical entities that can be used to develop new drugs [27,28].

**Figure 1:**
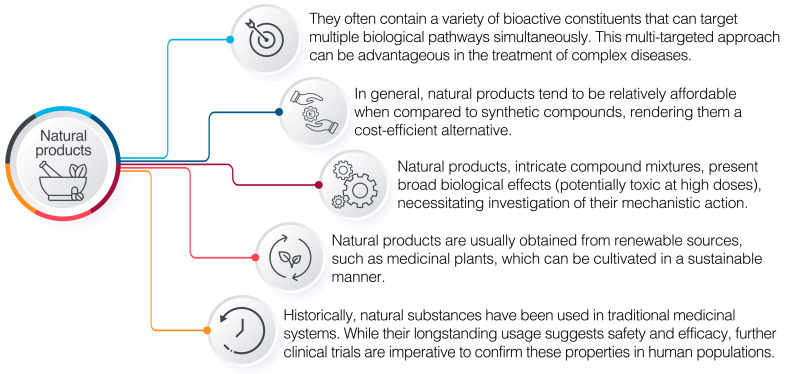
Key aspects of the use of natural products in pharmaceutics.

Many natural products have been successfully developed into drugs, including paclitaxel, derived from the bark and needles of *Taxus brevifolia* and used to treat various types of cancer and artemisinin, derived from sweet wormwood and used to treat malaria and many others. In addition, natural products have been used as lead compounds to develop synthetic analogues with improved potency, pharmacokinetics, and safety profiles [29]. Figure 1 illustrates the key aspects of the use of natural products in pharmaceutics.

Despite the potential benefits of natural products, there are also challenges associated with their use in pharmaceuticals. Natural products are often complex mixtures of compounds (extracts) exhibiting a wide range of effects on biological systems, necessitating clarification of their underlying mechanisms of action. Investigating natural products and phytochemicals presents a significant challenge due to their inherent complexity. Additionally, when these compounds are isolated from plants or microorganisms, they may undergo modifications that reduce or remove their therapeutic properties. Furthermore, natural products can vary in quality and potency depending on geographic location, climate, and cultivation practices [28,30,31].

Researchers use various strategies to overcome these challenges, including innovative extraction techniques and prefractionation [28] and standardization techniques. These approaches (such as semi-bionic extraction, molecular distillation, ultrasonic-assisted extraction, supercritical fluid extraction, and membrane separation) have yielded superior lead candidates for drug development [32,33]. These methods have been proven effective in isolating compounds from various sources. Additionally, advances in analytical techniques (i.e., high-performance liquid chromatography–mass spectrometry, nuclear magnetic resonance spectroscopy, etc.) for stability testing and quantification, as well as computational algorithms, have facilitated the synthesis of natural compound analogues for medicinal chemistry [26,34,35]. Employing -omics technologies can facilitate the investigation of compound combinations’ impact on cellular genes and proteins.

In addition, the advancement of biological constructs, including organoids and microfluidic systems, facilitates the comprehensive evaluation of substances on cellular and tissue samples. Using computational software to design, synthesize, and predict biological properties of new compounds derived from plant extracts will contribute significantly to drug discovery efforts [36,37]. While challenges are associated with their use, modern technologies contribute to overcoming these obstacles and unlocking the full potential of natural products in pharmaceutics.

## 2. Approach of the Review

This review aims to summarize the existing literature on using natural products and compounds to treat xerostomia. An exhaustive literature search was conducted utilizing the electronic databases of MEDLINE/PubMed, SCOPUS, and Google Scholar. This facilitated access to a vast array of original research articles in the English language, either previously published or categorized as “in press” within peer-reviewed academic journals. The keywords used included “xerostomia”, “natural products”, “artificial saliva”, “salivary stimulation”, “xerostomia drug formulations”, and “dry mouth drug formulations”. All articles published up to April 2023 were assessed in the preliminary phase without imposing additional constraints on publication type. The vital role of natural products in advancing diverse management strategies of xerostomia is undeniable, as evidenced by the number of studies encompassing various natural compounds in the disease management section. The results of this review aim to a better understanding of the potential role of natural products and compounds in managing xerostomia and guide future research in this area.

## 3. Natural Products Used for the Treatment of Xerostomia

Despite the growing number of literature reports on natural products and compounds for xerostomia treatment, the evidence remains scattered, with studies varying in methodology, quality, and outcomes. The subsequent sections present a comprehensive overview of the relevant studies, which are categorized into two primary groups: (i) in vivo studies and (ii) clinical trials.

### 3.1. In Vivo Studies

Apigenin, *Ixeris dentata* (Thunb.) Nakai *[Asteraceae]* (IXD) and *Lycium barbarum* L. represent naturally occurring plants that have been investigated for their potential therapeutic utility in addressing the symptoms of xerostomia in rat and diabetic mouse models (Table 1).

#### 3.1.1. Apigenin

Apigenin, a natural flavone found in various vegetables, fruits, herbs, and plant-based beverages, has been studied for its antidiabetic, anticancer, anti-inflammatory, and antimicrobial properties. Its administration is deemed non-toxic even in substantial quantities, and its extraction from natural origins is relatively straightforward. A recent study of Wei et al. (2022) reported the influence of apigenin therapy on xerostomia and the signaling compounds implicated in this disorder [2].

Aquaporin-5 (AQP5) proteins play a vital role in the pathophysiology of xerostomia. Studies revealed an escalation in estrogen receptor alpha (Erα)/AQP5 protein expression in apigenin-treated human submandibular gland (HSG) cells and the submandibular gland in ovariectomized (OVX) mice, suggesting the potential of apigenin to stimulate ERα/AQP5 signaling within the salivary glands.

Estrogen deficiency following menopause is associated with various health conditions, encompassing xerostomia, osteoporosis, diabetes, and other inflammatory diseases. Hormone replacement therapy (HRT) has been documented to augment salivary secretion in postmenopausal women. Yet, it also carries the possibility of unfavorable side effects, such as the progression of endometrial and breast cancers. Phytoestrogens, which are plant-derived dietary elements exhibiting structural similarity to estrogen (E2), can provoke estrogenic responses through their interaction with estrogen receptors.

The docking investigation showed analogous binding sites for apigenin and E2 on ERα, suggesting apigenin functions as an E2 mimic. It has been reported that apigenin therapy can mitigate the bone loss induced by estrogen deficiency in OVX mice, hinting at its potential to alleviate diseases related to estrogen deficiency, including xerostomia.

In that study, apigenin treatment significantly increased ERα and AQP5 expression in HSG cells in vitro and in the submandibular gland of OVX mice. Increased AQP5 expression and activity were observed in ERα-overexpressing HSG cells treated with apigenin. However, in ERα-deficient HSG cells, apigenin did not enhance AQP5 expression and activity, which suggests an ERα-dependent effect of apigenin on AQP5 expression and activity. Both apigenin and E2 treatment boosted the salivary secretion index and reduced water intake in OVX mice [2].

#### 3.1.2. *Ixeris dentata* (Thunb.) Nakai Extract

Bhattarai et al. (2018) studied the effect of *Ixeris dentata* (Thunb.) Nakai *[Asteraceae]* (IXD) ethanolic extract for treating xerostomia on 40 Sprague Dawley diabetic rats. Rats were divided into four equal groups (Group 1: Control rats treated with water; Group 2: Control rats treated with the IXD extract; Group 3: Diabetic rats treated with water; Group 4: Diabetic rats treated with the IXD extract) (IXD extract dose: 100 mg/kg body weight).

Results revealed that diabetic rats treated with IXD showed lower saliva and blood glucose concentrations, improved total body weight, and higher expression of salivary α-amylase. Furthermore, IXD extract increased the expression of AQP5 and amylase in the submandibular gland, suggesting a potential target to improve xerostomia. Moreover, diabetic rats treated with IXD extract showed limited glucose-regulated protein 78 GRP78 secretion in saliva, suggesting a possible inhibitory action against stress. IXD extract was observed to increase the salivary flow rate and α-amylase expression in diabetic rats. The study also found that the IXD extract reduced oxidative stress exacerbated by hyperglycemia, maintained sufficient salivary enzyme secretion, and increased amylase secretion in hyperglycemic conditions [38].

In a subsequent investigation undertaken by this research group, the effect of IXD extract on age-related xerostomia was investigated in a study utilizing 85 adult male Sprague Dawley rats. The rats were randomly assigned to eight distinct groups designated for young and ageing models (n = 10–15 per group). The rats were provided unrestricted access to tap water and standard food pellets throughout the experimental duration. Ageing rats (20 months old) and young rats (2 months old) received either water or IXD extract treatment via oral gavage for a period of eight weeks.

The findings revealed that ageing rats’ submandibular gland weight was significantly greater than young rats (*p* < 0.05). No differences were observed in whole saliva volume between young and ageing rats. In ageing rats, administering IXD extract at 100 mg/kg dosage led to a significant increase in total saliva compared to younger rats under the same treatment conditions (*p* < 0.05). Histological assessments revealed significantly reduced acinar cells and enlarged striated ducts within the submandibular glands in older rats. In contrast, juvenile rats displayed dense acinar cells and well-maintained striated ducts. These findings suggest that IXD extract can potentially augment saliva production in ageing rats, which may alleviate xerostomia symptoms. The results imply that IXD extract could potentially enhance salivary flow in ageing rats by modulating oxidative stress and the unfolded protein response [39].

#### 3.1.3. *Lycium barbarum* L.

*Lycium barbarum* L. polysaccharides (LBP) are extracted from *Lycium barbarum L.* (using H_2_O). They are used in traditional Chinese medicine and have been investigated for its diverse therapeutic effects, including immune regulation and anti-inflammatory responses. The immunomodulatory and anti-inflammatory attributes of LBP display variance in relation to dosage. A study by Wang et al. (2021) examined the therapeutic effectiveness and underlying mechanisms of LBP in primary Sjögren’s syndrome (pSS) utilizing NOD mice, which were randomly segmented into four groups and treated over a span of 12 weeks.

Findings demonstrated that a low dosage of LBP enhances salivary flow rates and diminishes inflammation within the SMGs, contributing favorably to the progression of pSS in NOD mice. The potential mechanism propelling LBP’s immunomodulatory and anti-inflammatory actions was explored by assessing the differentiation of T cell subsets influenced by LBP treatment. Data indicated a vital role for LBP.L in modulating CD4+ T cell differentiation in vivo by augmenting regulatory T cell ratios and decreasing the counts of pro-inflammatory cells, encompassing Tfh, Th1, and Th17 cells.

Employing an innovative human disease model, the research pinpointed a potential function for LBP in reinstating immune equilibrium and homeostasis, thereby hindering the advancement and emergence of pSS. Nevertheless, the study encountered limitations. Like most traditional Chinese medications, LBP is a composite substance of multiple monosaccharides and amino acids. Further exploration is needed to identify the precise active component accountable for LBP’s immunomodulatory effects. The wide-ranging biological activities of LBP are dose responsive, with moderate or high doses triggering humoral and cellular immunity and low doses suppressing the production of inflammatory factors, thereby inducing anti-inflammatory and immunomodulatory responses. Establishing the optimal LBP dosage for treating autoimmune diseases presents a considerable challenge.

Furthermore, the study did not investigate the specific targeted molecules, genes, and signaling pathways involved. While the potential for clinical translation of LBP as a pSS therapeutic remains exciting, further evaluation is necessary [40].

### 3.2. Clinical Trials

Various natural substances and compounds have been explored for their prospective application in the treatment of xerostomia. These include but are not limited to green tea, thyme honey, lycopene, ginger, aloe vera, chamomile, linseed, and coconut oil. In the subsequent sections, a total of 17 studies are presented in detail (Table 2), at which clinical trials have been conducted to assess the effectiveness of these diverse natural products in mitigating the symptoms associated with xerostomia.

#### 3.2.1. *Camellia sinensis* (L.) (Green Tea)

In a double-blind, placebo-controlled, randomized study, researchers evaluated the effectiveness of MighTeaFlow (MTF), a natural formula composed of two plant extracts, one of which is green tea catechins, in treating xerostomia. The participants ranged between 21 and 74 years, and 91% had tried over-the-counter medications or sialogogues for xerostomia before the study. No significant adverse events were reported during the trial.

Participants (60 patients) were divided into two groups: Group A (intervention) using the MTF lozenge and Group B (control) using a placebo lozenge (containing the other formulation ingredients and 500 mg xylitol, but without the MTF formula). The study found a significant difference in improvement in stimulated whole saliva flow rate (SWSFR) between the two groups over the entire study. The intervention group experienced a 1.5-, 1.7-, and 2.1-fold increase in SWSFR relative to baseline at weeks 1, 4, and 8, respectively, while the placebo group showed no meaningful change.

Similarly, there was a significant difference in unstimulated whole saliva flow rate (UWSFR) improvement between the two treatments over the entire study. Participants in the intervention group experienced a 3.3-, 3.1-, and 3.8-fold increase in UWSFR relative to baseline at weeks 1, 4, and 8, respectively. In contrast, the placebo group showed no meaningful change in UWSFR throughout the trial period.

Nevertheless, the QoL evaluation exhibited no substantial variance in the transformation between the two treatment groups throughout the study. Both groups experienced significant QoL improvements compared to the baseline at visits 2, 3, and 4. The incongruity between the advantageous impact on objective flow rates and subjective QoL indicators still needs exploration.

In vitro and in vivo studies indicate green tea polyphenols (GTPs)/(-)-epigallocatechin-3-gallate (EGCG) as potential natural agents for xerostomia management, potentially delaying salivary dysfunction through molecular mechanisms. Researchers highlighted EGCG’s role in suppressing autoantigens, influencing epithelial cell proliferation, and modulating antioxidant enzyme expression in salivary glands.

In summary, the MTF formula demonstrated a statistically significant elevation in both SWSFR and UWSFR, offering an objectively viable strategy for managing xerostomia devoid of detrimental effects. However, the MTF formula and the placebo groups did not significantly differ in QoL measures. To evaluate the effectiveness of the MTF formula, further research is necessary, encompassing larger sample sizes and trials involving patients afflicted with head and neck cancer or Sjögren syndrome. The underlying mechanism behind the synergy obtained by combining ingredients in the proprietary formulation also needs further investigation [41].

#### 3.2.2. *Thymus* L. (Thyme Honey)

The study investigated the effectiveness of thyme honey in treating xerostomia in head and neck cancer patients undergoing radiotherapy. The sample consisted of 72 patients aged 32 to 93 years, equally divided into an experimental and a control arm, with no significant difference in age, gender, education, or cancer type. The intervention arm received thyme honey, while the control arm received saline.

First, it should be noted that the safety and tolerability of thyme honey were excellent, with no reported side effects and high adherence to the protocol. Thyme honey demonstrated efficacy in lessening or maintaining the severity of xerostomia throughout the research period, with a gradual enhancement observed until the seventh week. Conversely, xerostomia in the control group followed a linear worsening trajectory until the fifth week, after which it stabilized. Half a year post intervention, patients in the experimental group displayed significant amelioration in their xerostomia level relative to those in the control group.

Thyme honey also positively affected other symptoms, such as pain, taste loss, and dysphagia (difficulty swallowing). Patients in the intervention arm experienced a lower prevalence of severe xerostomia and pain, and a smaller percentage required soft foods or liquids for feeding. The intervention’s positive impact on these symptoms was found to be statistically significant.

QoL and general satisfaction were assessed using questionnaires, revealing that the intervention arm had significantly higher QoL and satisfaction scores at months one and six after the intervention, with substantial effect sizes.

In conclusion, the study supports the claim that thyme honey can effectively and safely manage xerostomia in head and neck cancer patients during and after radiotherapy. The intervention showed significant improvements in xerostomia, pain, taste loss, and dysphagia, as well as QoL and general satisfaction because honey has the ability to stimulate the gustatory system [42]. However, despite the absence of reported adverse effects within the six-month observation period of this study, it is crucial to acknowledge concerns regarding honey’s high free sugar content. Previous studies have linked such high sugar content to radiogenic caries, a significant health concern [57,58]. Therefore, it is imperative to conduct more comprehensive and targeted research in this area to understand fully the potential risks and implications associated with the consumption of honey, particularly in relation to oral health, caries development, and the potential impact on treatment outcomes.

#### 3.2.3. Fermented Lingonberry Juice

A study was conducted to investigate the effects of FLJ (lingonberry is the common name of *Vaccinium vitisidaea* L.) on the treatment of xerostomia. The participants included 21 middle-aged Scandinavians who used FLJ mouthwash (10 mL daily for 30 s) for 6 months, followed by a 6-month washout period without the FLJ mouthwash regimen. Salivary parameters were measured at three time points: 0, 6, and 12 months.

The findings indicated that FLJ mouthwash had a positive impact on all five salivary metrics, with statistically significant enhancements observed throughout the duration of the study. The saliva flow rate at rest elevated from low to standard levels during the FLJ phase and remained steady during the washout phase. Resting salivary pH progressively increased during the trial, and the flow rate of stimulated saliva enhanced during the FLJ phase, continuing to increase during the washout phase. The buffering capacity escalated from near-minimal values to mean values during the FLJ phase, maintaining normal levels throughout the washout period.

Subjective symptoms of oral dryness reduced during the period of FLJ mouthwash usage and sustained a lower level during the washout phase compared to the commencement of the study. These symptoms displayed a negative correlation with resting and stimulated saliva flow rates and resting saliva pH, indicating that an elevation in these parameters decreased the sensation of oral dryness.

Importantly, four participants did not adhere to the prescribed instructions, and their buffering capacities did not display statistically significant alterations. Participants reported no erosive lesions, mucosal irritation, or adverse effects from using FLJ mouthwash.

The study demonstrated that FLJ mouthwash was beneficial in maintaining safe saliva pH and improving salivary parameters, making it a potential natural remedy for xerostomia. The exact mechanisms behind these effects have yet to be precisely discovered. Still, possible mechanisms may involve the induction of salivary gland secretion and antioxidant, anti-inflammatory, anti-proteolytic, and antimicrobial effects of FLJ’s complex polyphenol composition [43].

#### 3.2.4. *Cocos nucifera* L. (Coconut Oil)

Quimby et al. (2020) assessed the feasibility of using coconut oil as a treatment for xerostomia in 30 patients (20 male and 10 female) who had undergone radiation therapy for head and neck cancer. Participants were instructed to apply coconut oil before meals and before going to bed by coating their mouth with the product. They were given the freedom to adjust the frequency and amount of coconut oil used based on what provided the most relief for their symptoms of xerostomia. No notable relations were discovered between initial demographic variables and the probability of persisting coconut oil usage beyond the initial fortnight. The xerostomia-related XeQoLS was implemented at the onset and the three-month follow-up mark.

The study duration was two weeks, during which 73.1% of patients utilized coconut oil consistently. The average total period of coconut oil application was 16 days. The daily usage was typically three times, with an average quantity of 5 mL of coconut oil per usage. The most frequent triggers for use were mealtimes and before sleep. A total of 12 participants (41.4%) continued using coconut oil beyond the recommended 2-week period.

Although a considerable portion of participants continued to use coconut oil beyond the designated trial period, no overall difference in XeQoLS scores was noted before and after the treatment. In fact, further examination of the pre- and post-treatment XeQOLS scores revealed that 13 patients (50%) exhibited significantly increased (worse) XeQOLS scores during the follow-up period. No adverse reactions linked to coconut oil use were reported during the study. This suggests that while some patients may find intermittent coconut oil usage beneficial, the overall impact on xerostomia might be minimal.

The study’s limited sample size restricts the ability to conclusively determine the effectiveness of coconut oil in improving xerostomia among head and neck cancer patients. The alleviation of xerostomia using coconut oil may be attributed to its capacity to form a protective layer in the oral cavity, which helps maintain mucosal surface hydration. Nonetheless, the feasibility of coconut oil as a therapeutic intervention for radiation-induced xerostomia in this patient demographic was successfully evaluated, and the parameters of coconut oil use deemed effective by study participants were delineated [44].

#### 3.2.5. Lycopene-Enriched Olive Oil

This study explored the effectiveness of lycopene-enriched virgin olive oil (*Olea europaea* L.) as a treatment for xerostomia. A total of 60 patients with drug-induced xerostomia participated in a randomized, double-blinded, placebo-controlled clinical trial, with 30 in the treatment group (Group A) and 30 in the placebo group (Group B). The treatment group received lycopene-enriched virgin olive oil (300 mg/L) in a spray container, while the placebo group received a placebo. The spray was administered to the mouth, with a volume of 1.5 mL, and then swallowed. Patients were given instructions to use the medication three times per day and were not allowed to use any other gel or mouthwash during the study period. The study lasted 12 weeks, during which no adverse effects were reported.

Sialometry draining test results showed an increase in non-stimulated saliva flow for both groups, with a statistically significant improvement in the treatment group (*p* = 0.001) and the placebo group (*p* = 0.003). Analysis of VAS (visual analogue scale) symptom scores revealed significant improvements for specific items in both groups, indicating that the treatment group experienced more significant improvements in some symptoms than the placebo group. When examining oral quality-of-life data, the treatment group showed significant differences between day 0 and 12 weeks of treatment (*p* = 0.001), while no differences were observed in the placebo group (*p* = 0.10).

Despite the higher patient-evaluated benefits and satisfaction with the spray in the treatment group, the variance between the groups was not statistically substantial. The findings suggest that topical lycopene-rich olive oil may enhance symptoms of xerostomia, the rate of salivary flow, and oral quality of life. However, the statistical comparative analysis between groups revealed no significant difference between the treatment and placebo groups.

The present study suggests that topical lycopene-enriched virgin olive oil in spray form permits immediate contact with oral structures and is safe and well tolerated. However, it is unclear whether the spray itself or its active substance is responsible for the improvements observed in xerostomia-related symptoms. Future studies should assess the effectiveness of lycopene-enriched virgin olive oil in treating xerostomia caused by other factors, such as radiotherapy or Sjögren’s syndrome. Overall, administering lycopene-enriched virgin olive oil in spray form may improve oral quality of life, reduce symptoms, and be a novel therapeutic strategy for treating xerostomia [45].

#### 3.2.6. *Zingiber officinale* Roscoe (Ginger)

Mardani et al. (2017) investigated the effect of ginger herbal spray on dry mouth in 20 patients (aged between 49 and 69 years old) with type II diabetes. The control subject for each patient was the patient themself. Each participant completed a questionnaire on three separate occasions (before treatment, following treatment with a placebo, and post-drug administration). The Schirmer test was also conducted to assess the patients’ salivary flow. The investigational drug and placebo were formulated as oral sprays containing herbal ginger extracts (containing ginger ethanolic extract, 1/3 edible glycerin, and 1/3 distilled water.). The mean salivary volume significantly increased after applying the ginger plant spray (*p* < 0.001). The mean salivary volume post-medication treatment demonstrated a considerable difference compared to the mean salivary volume post-placebo treatment (*p* < 0.001) [46]. The study’s results support the idea that ginger effectively increases salivation and reduces xerostomia in patients.

Chamani et al. (2017) performed a randomized double-blind, parallel clinical trial of ginger usage in patients with post-radiotherapy xerostomia, involving 61 patients who have prescribed either a ginger capsule (Zintoma, Goldaru Company, Tehran, Iran) or a placebo to assess the treatment of xerostomia, or dry mouth. Using the VAS, patients were questioned about xerostomia before receiving ginger treatment or a placebo. On day 14, the mean treatment effect was 33.7 ± 20.9 mm VAS score in the ginger group and 23.6 ± 17.3 mm VAS score in the placebo group.

The study demonstrated a borderline significant amelioration of xerostomia symptoms associated with ginger administration (*p* = 0.057). The severity of major xerostomia manifestations, such as difficulties with chewing, swallowing, and speech, altered taste ability, and oral burning sensation, were compared across both groups, revealing no notable disparities. On day 14, the impact of the treatment on their QoL and improvement of xerostomia symptoms was assessed. No significant variations were detected between the groups concerning improved xerostomia symptoms and QoL. Nevertheless, in response to one particular question about experiencing dry mouth throughout the day, 82% of patients in the ginger group and 62% in the placebo group reported an improvement, which is a difference of borderline significance (*p* < 0.100).

Some patients registered side effects following medication intake. In the ginger group, one patient encountered constipation, two reported vertigo and two experienced dyspepsia. In the placebo group, two patients had headaches, and two had dyspepsia. These side effects were minor, and it is plausible that other factors were responsible for these issues rather than ginger or placebo consumption.

In conclusion, this study presented a marginally significant improvement in xerostomia symptoms with ginger intervention. However, there were no significant differences between the groups regarding xerostomia symptom improvement and QoL, except for experiencing dry mouth throughout the day [47].

Badooei et al. (2021) conducted a triple-blind clinical study involving three groups, each consisting of 35 patients, to investigate the effects of ginger and aloe vera mouth rinses on xerostomia symptoms in individuals with diabetes. Participants in one group were given an aloe vera mouthwash, the second group utilized a ginger mouthwash, and the control group was provided with normal saline only. All mouth rinses were administered in 20 mL doses three times per day for a duration of 14 days consecutively. The severity and symptoms of xerostomia were assessed using a questionnaire and a VAS before and following the intervention.

Post intervention, the data revealed that those in the ginger and aloe vera groups reported a significantly lower incidence of xerostomia symptoms, including the need to drink water for swallowing dry food, reduced salivation, oral dryness upon awakening, dry mouth while traveling, and a sensation of burning in the mouth. The findings suggest that both ginger and aloe vera mouthwashes notably diminished all symptoms of xerostomia, with ginger producing the most pronounced effect, followed by aloe vera and then normal saline.

This study had some limitations, including patients using mouthwashes at home without direct supervision and completing questionnaires self reportedly, which may have led to inaccuracies [48].

Concerning the mechanism through which ginger augments salivary secretion, researches have substantiated that this effect is achieved by engaging parasympathetic activity on the postsynaptic M3 receptors and exerting a suppressive influence on presynaptic muscarinic auto receptors [59,60].

#### 3.2.7. *Aloe vera* (L.) Burm.f. and *Mentha* L. (Peppermint)

A triple-blind, two-group, randomized, placebo-controlled clinical trial was conducted using a convenience sample of 80 patients. Participants were randomly assigned to either an intervention or a placebo group. For five consecutive days, oral care was administered to patients in the intervention and placebo groups utilizing Veramin moisturizing gel (containing 100% A. vera jelly, 3% peppermint essential oil, carboxymethyl cellulose, 10% propylene glycol, and 0.1% potassium sorbate) and a placebo gel, respectively. This study aimed to assess the impact of a moisturizing gel comprising aloe vera and peppermint essential oil on xerostomia and oral health in intensive care unit (ICU) patients.

The results indicated that the gel (Veramin) significantly reduced mouth dryness and improved the patients’ oral health. More specifically, on the fifth day, the mean mouth dryness score in the intervention group was significantly lower than that in the placebo group (*p* = 0.0001). Veramin moisturizing gel effectively alleviated xerostomia, inhibited dental plaque formation, and enhanced oral health. Veramin gel’s effectiveness in relieving oral dryness is attributed to the unique properties of Aloe vera. With approximately 99% water content in the inner jelly of the Aloe vera leaf, it provides powerful moisturizing benefits. Additionally, the presence of mucopolysaccharides helps retain moisture within the mucosa. Moreover, Aloe vera demonstrates anti-inflammatory effects by inhibiting the cyclooxygenase pathway and reducing prostaglandin E2 levels. Due to these advantageous characteristics, aloe vera has been employed in treating various conditions, including lichen planus, burning mouth syndrome, and mucositis.

The study encountered limitations, including a brief follow-up period, and being conducted in a singular healthcare setting. Nonetheless, Veramin moisturizing gel was proposed as efficacious in significantly reducing xerostomia, preventing dental plaque development, and ameliorating oral health [49].

#### 3.2.8. Glucosylceramide Extracted from *Ananas comosus* (L.) Merr. (Pineapple)

Murakami et al. (2019) performed a double-blind randomized cross-over trial to assess the effects of oral intake of Glucosylceramide extracted from pineapple (GCP) on oral moisture and xerostomia symptoms. Generally, it is reported that the consumption of GCP has been found to have positive effects on ceramide synthesis and cell turnover in the lingual mucosa. As a result, an increase in oral moisture levels is anticipated, leading to an improvement in the condition of xerostomia. This treatment option for xerostomia could serve as a natural alternative to commercial oral moisturizers and chemical-based products. The study involved a comparison between GCP tablets and placebo tablets. The GCP tablets were formulated with 1.2 mg of glucosylceramide. A total of 16 participants were randomly selected and evaluated between July 2016 and January 2019. The participants were given instructions to consume tablets from the initial test sample once per day, specifically after breakfast, for a continuous period of two weeks. No side effects caused by GCP or placebo tablets were observed. The study had a cross-over design, with an eight-week study period including a two-week intake and a four-week washout period. The washout period was sufficient to avoid carryover effects.

The results indicated that oral moisture significantly increased, and the VAS value for mouth dryness was significantly improved after GCP tablet intake compared to placebo tablet intake. There were no significant differences in the number of fungiform papillae before and after taking GCP tablets and placebo tablets. The study had some limitations, including a relatively low number of subjects in each group (n = 8) and relatively high oral moisture levels at baseline. The participants were mainly elderly individuals with hypertension as the primary underlying disease, taking antihypertensive drugs, which is a risk factor for xerostomia [50].

#### 3.2.9. *Linum usitatissimum* L. (Linseed)

Andersson et al. (1995) explored the impact of linseed extract, also known as mucilage, on symptoms of dry mouth in patients experiencing reduced salivation as a side effect of radiation treatment for malignant conditions in the head and neck area. The study included 20 patients (9 female, 11 male) with serious hyposalivation and previously attempted various saliva replacement therapies without marked success. This study tested two saliva substitutes: (i) Salinum^®^, a linseed extract primarily composed of water-soluble polysaccharides (approximately 54% non-starch polysaccharides and 9% protein), sourced from the Tadorna variety and (ii) MAS-84, a product based on sodium carboxymethyl cellulose.

The findings illustrated that both tested preparations provided beneficial effects on symptoms of dry mouth, with patients reporting relief from these symptoms when utilizing the linseed extract as a saliva substitute. The intensity of dry mouth symptoms was analyzed using a VAS. The notable disparity in response patterns between Salinum and MAS-84 corroborates patient preference for Salinum, which appears to ameliorate some functional deficiencies associated with reduced salivation from minor salivary glands. When patients utilized Salinum, plaque and gingival indices significantly decreased, suggesting enhanced oral hygiene practices and/or augmented mucosal resilience to mechanical or chemical factors.

The mean efficacy duration of Salinum was observed to be approximately 60 min, while that of MAS-84 was half as long, at 30 min. A significant contrast in the quantity of Salinum and MAS-84 used was identified, with nearly triple the volume needed for the MAS-84 formulation. The lesser quantity of Salinum needed to mitigate dry mouth symptoms benefits patients by lessening the cost and addressing practical considerations associated with the management of the saliva substitute.

There was a substantial variation in relief from dry mouth symptoms across patients and the two examined formulations, which could potentially be attributed to diverse levels of structural damage to the salivary glands and varying deficits in saliva. Linseed extract, particularly the Tadorna variety, was selected due to its high viscosity, biofilm formation capability, and resilience to shear forces. Examining different linseed varieties has disclosed variations in viscosity, polysaccharide makeup, and protein content, with the mucilage primarily consisting of water-soluble polysaccharides and having strong water binding capacity [51].

#### 3.2.10. *Matricaria chamomilla* L. (Chamomile) and *Linum usitatissimum* L. (Linseed)

The efficacy of a combined chamomile and linseed saliva substitute in relieving xerostomia, or dry mouth, in older participants was evaluated by Morales-Bozo et al. (2017) in a double-blind, randomized, cross-sectional clinical trial. The motivation behind this study stemmed from the fact that linseeds are abundant in proteins, unsaturated oils, insoluble fibers, and soluble fibers or mucilages. When linseeds come into contact with moisture, their lining cells release significant amounts of mucilage, creating a gelatinous capsule around them. This water-soluble mucilage contains polymers with high molecular weight and viscoelastic properties similar to the salivary mucins responsible for hydrating the oral mucosa. Additionally, dry chamomile flowers are of interest due to their content of essential oils and flavonoids with sedative, antispasmodic, anti-inflammatory, and antimicrobial effects. In clinical trials involving head and neck cancer patients undergoing radiation or chemotherapy, and experiencing irritation of the oral mucosa, the administration of chamomile mouthwash offered relief from oral discomfort. Furthermore, the patients’ clinical signs of mucositis disappeared within the initial week of using the mouthwash [61].

Using a VAS scoring system, the researchers assessed the decrease in symptoms before and after using the saliva substitutes. The chamomile and linseed saliva substitute (prepared as aqueous extracts from chamomile and linseed seeds) positively affected four out of five xerostomia symptoms, while the conventional saliva substitute only impacted two of them. Additionally, participants were asked to evaluate their sense of relief from xerostomia symptoms when using each substitute on a scale from 1 to 10. The chamomile and linseed saliva substitute provided greater relief for three of the five symptoms than the conventional substitute.

However, the study has some limitations. The short follow-up time makes it difficult to determine the long-term effectiveness of the chamomile and linseed saliva substitute. A more extended evaluation period would be necessary to confirm the prolonged effectiveness of the herbal substitute. Another limitation of the study is the sample composition, mainly older women with xerostomia due to pathologies that cause the condition. Of the participants, 54.1% (40 out of the 74) had autoimmune pathologies, such as Sjögren’s syndrome, rheumatoid arthritis, and systemic lupus erythematosus, which are associated with xerostomia and are more frequently found among women. The researchers could not definitively rule out a gender difference in the effectiveness of the chamomile and linseed saliva substitute [52].

#### 3.2.11. *Hibiscus sabdariffa*

Levrini et al. (2020) assessed the efficacy of Aqualief™ (Helsinn Healthcare SA, Lugano, Switzerland) in treating xerostomia, or dry mouth, in patients contacting a randomized, placebo-controlled, double-blind trial. Aqualief™ contains two key ingredients, carnosine and karkadé (*Hibiscus sabdariffa*), which were selected and mixed with normalizing saliva pH and increasing saliva buffering activity. These parameters are often impaired in xerostomia patients, leading to acid-induced enamel and dental erosion and promoting the growth of aciduric bacteria.

Aqualief™ was found to normalize saliva pH to a neutral value and significantly increase the saliva flow rate in xerostomic patients. After six days of treatment, saliva pH was increased toward a neutral value, and the saliva flow rate was increased by almost 60%, compared to the basal value. This improvement was more than three times greater than that achieved with a placebo, which only increased resting salivation by 19%.

The ability of Aqualief™ to normalize saliva pH could be attributed to the presence of carnosine, which stimulates carbonic anhydrase activity. Still, other mechanisms may also play a role, such as increased saliva production, stimulation of carbonic anhydrase activity, or changes in oral microbiota composition. The salivation-promoting effect of Aqualief™ could be due to its balancing effect on pH or a direct impact of carnosine and/or karkadé components.

However, this study had limitations: xerostomic patients with hyposalivation could not be recruited to test Aqualief™ because preliminary studies found that a minimum resting saliva flow of 0.2 mL/min was needed to dissolve the tablet. A new water-based formulation has been developed for xerostomic participants with hyposalivation and will be tested in the future. Additionally, the duration of the effects after discontinuation of treatment has not yet been investigated [53].

#### 3.2.12. *Malva sylvestris* L. and *Alcea digitata* (Boiss.)

*Malva sylvestris* L. and *Alcea digitata* (Boiss.) Alef. have been employed in traditional Persian medicinal practices for their cough suppressant, antioxidant, expectorant, anti-inflammatory, antimicrobial, and laxative properties. A study conducted by Ameri et al. (2016) assessed the effectiveness of an herbal mixture containing *Malva sylvestris* and *Alcea digitata* in treating radiation-induced xerostomia in a cohort of 62 patients (41 males and 21 females), contrasting it with Hypozalix (control treatment) in a randomized clinical trial. The test group (average age 51.5) received the herbal formulation, while the control group (average age 50.3) was treated with Hypozalix.

The herbal mixture and Hypozalix both positively affected symptoms of dry mouth. A comparative analysis between the test and control groups revealed a substantial reduction in the VAS score for the test group after four weeks, relative to the control group. This result endorses the subjects’ preference for the herbal mixture. In the test group, three subjects exhibited a decrease in dry mouth severity, while no change was noticed in the control group. However, the change in dry mouth severity was not statistically significant between the groups. The modifications in the dryness grade at the two-week mark suggest that additional research involving a larger sample size and extended duration is necessary to ascertain the efficacy of the herbal mixture in enhancing dry mouth severity.

A significant degree of variability was noted in the reduction of dry mouth symptoms among individuals in both cohorts. This variation in response could potentially be ascribed to distinct patterns of structural injury to the salivary glands, culminating in varying extents of saliva insufficiency. Studies suggest that medicinal mucilage could alleviate dry mouth through two potential mechanistic pathways. Primarily, it might function as a salivary secretion analogue, providing lubrication and protection to mucous membranes. The secondary mechanism, rooted in traditional Persian medicinal knowledge, attributes the effect to the inherent properties of the plant. The medicinal mucilage is purported to possess hydrating and mild cooling properties, which could counterbalance dryness [54].

#### 3.2.13. Vitamin C/E Complex

In a study conducted by Chung et al. (2016), the potential preventive efficacy of a complex supplement of vitamins C and E in counteracting xerostomia instigated by radiotherapy (RT) was explored in patients diagnosed with head and neck cancer. This was done using a prospective, randomized, placebo-controlled, double-blind study design. The study comprised 45 participants, divided into two groups: the experimental group (n = 25), which was given antioxidant supplements of 100 IU of vitamin E and 500 mg of vitamin C twice a day during RT, and the control group (n = 20) which was given a placebo identical in appearance. To compare the severity of xerostomia between the two groups, evaluations were done using patient-reported xerostomia questionnaires, observer-rated xerostomia scores, and salivary scintigraphy before RT and one- and six-months post RT.

At the six-month post-RT checkpoint, the experimental group demonstrated a superior enhancement in both the xerostomia questionnaire scores and the observer-rated xerostomia scores compared to the scores recorded at the one-month post-RT checkpoint (*p* = 0.007 and 0.008, respectively). Conversely, the control group showed no substantial alterations between one- and six-months post RT. Salivary scintigraphy disclosed no differences in maximal accumulation or ejection fraction parameters between the two groups. However, compared to the control group, the experimental group retained significantly superior oral indices at the prestimulatory (*p* = 0.01) and poststimulatory (*p* = 0.009) phases at one-month post RT. At the end of the study, there was no observable difference in overall survival and disease-free survival between the two groups.

The findings of this study imply that short-term supplementation with an antioxidant complex of vitamins E/C may offer protection against RT-induced xerostomia in patients with head and neck cancer. In particular, the data obtained from experiments conducted on cancer cells and animal models demonstrates that antioxidants exhibit a preference for safeguarding normal cells rather than cancer cells against oxidative damage induced by radiation. As a result, employing antioxidants could potentially mitigate the adverse effects related to radiation therapy. Moreover, at one-month post RT, the experimental group displayed significantly improved oral indices during both prestimulatory and poststimulatory stages compared to the control group, implying the possible advantages of antioxidant supplementation during RT [55].

#### 3.2.14. *Plantago ovata* Forssk.

In a double-blind, randomized, controlled crossover trial, Hasheminasab et al. (2020) evaluated the efficacy of a *Plantago ovata*-based herbal compound in prophylaxis and management of oral mucositis among breast cancer patients undergoing chemotherapy. The clinical outcomes, including the degree of mucositis, pain severity, xerostomia grade, and quality of life, were evaluated across screening, placebo, and treatment groups. With the screening cycle displaying the highest symptom severity, followed by the placebo, and the treatment group, the latter displayed the highest quality of life scores.

Significant clinical improvements were observed in the treatment group administered with the *Plantago ovata* husk. The degree of mucositis (*p* = 0.014), pain severity (*p* = 0.006), and xerostomia grade (*p* = 0.046) significantly decreased, while quality of life improved (*p* = 0.013). Similar improvements were noted when comparing the placebo group with the screening cycle (*p* < 0.05), and minimal side effects were reported.

Results show superior outcomes for patients given the herbal compound and oral care protocol compared to placebo and oral care protocol, as seen across all clinical outcomes one-week post-adriamycin initiation. This suggests that a mouthwash containing *Plantago ovata* hydrocolloid and vinegar can reduce oral mucositis (OM) severity and complications. Notable improvements in OM between the screening cycle and placebo group underscore the effectiveness of the oral care protocol in OM prevention and treatment. Although the placebo effect was considered, the significant role of the oral care protocol in these improvements indicates its value as a first-line option for OM management, possibly alongside other treatments.

Furthermore, the topical application of the *Plantago ovata* hydrocolloid and vinegar herbal compound contributed to the reduction in OM severity and complications, demonstrating its potential as a natural adjunct therapy for managing OM. The rationale behind these observations was derived from references present in the literature, which indicate that specific plants used in Persian medicine for treating various oral conditions are referred to as “loab plants” and primarily contain mucilage. Mucilage is a polysaccharide structure that forms a gel upon exposure to water and subsequent hydration. Recent studies have highlighted that certain compounds extracted from mucilage polysaccharides possess diverse pharmacological activities, such as anti-inflammatory, antioxidant and anti-allergic effects, while also offering protection to mucous membranes. These “loab plants” include *Plantago ovata* [56].

#### 3.2.15. Pilocarpine

Pilocarpine is a parasympathomimetic compound that primarily functions as a non-specific muscarinic acetylcholine receptor agonist while also possessing mild beta-adrenergic activity. This tertiary alkaloid is derived from plants of the *Pilocarpus* genus, particularly found in the leaves of *Pilocarpus microphyllus* and *Pilocarpus jaborandi*.

As the most prevalent medication for the treatment of Sjögren’s syndrome and relief of radiation-induced xerostomia symptoms, pilocarpine has gained approval from the U.S. Food and Drug Administration (FDA). Numerous studies have demonstrated the efficacy of administering pilocarpine in managing xerostomia, which can be divided into systemic and topical administration [12]. Pilocarpine stimulates natural salivation by binding to the muscarinic acetylcholine receptor 3 (M3R) located in the acinus cells of the salivary glands. The muscarinic acetylcholine receptors (mAChR) consist of five distinct subtypes, M1–M5, and play a crucial role in regulating various fundamental functions of both the central and peripheral nervous systems. Pilocarpine has the ability to activate all five muscarinic receptor subtypes, but its therapeutic effects primarily stem from its interactions with M3R [62].

##### Systemic Administration of Pilocarpine

Systemic administration of pilocarpine is mainly performed via oral tablets, such as Salagen^®^ (5 mg pilocarpine HCl), approved for radiation-induced xerostomia treatment in Europe and the USA. The recommended dosage ranges from 2.5 to 10 mg, taken orally three or four times daily, with adjustments made depending on patient response. The drug’s central and peripheral muscarinic effects manifest within 20 min of ingestion, and its elimination half-life is approximately 0.76–1.3 h.

Randomized, placebo-controlled trials have confirmed the efficacy of systemic administration of pilocarpine in increasing resting and stimulated salivary flow, decreasing subjective oral dryness, and improving chewing and speaking abilities in patients with Sjögren’s syndrome and post-radiation xerostomia. Evidence suggests that administering oral pilocarpine before and during radiotherapy may lead to longer-lasting results.

Despite its efficacy, the administration of systemic pilocarpine has been linked to a range of adverse reactions. These include hyperhidrosis, nausea, emesis, diarrhea, rhinitis, cephalgia, thoracic pain, abdominal contractions, vertigo, palpitations, chilling, flu-like syndromes, augmented urinary frequency, and enhanced lacrimation. These side effects can be anticipated due to the pharmacological influence of pilocarpine on exocrine glands, such as the sudoriferous, salivary, lacrimal, pancreatic, and intestinal glands. Pilocarpine is not advisable for patients suffering from gastric ulcers or uncontrolled asthma. Additionally, the drug’s potential cardiovascular impacts must be considered [12].

The pronounced systemic side effects of oral pilocarpine formulations may result in poor tolerance and compromised patient compliance with the treatment. Hyperhidrosis is the most reported adverse effect and exhibits a dose-responsive trend. In a research study conducted by Rieke et al. (1995), the recommended therapeutic dosage was determined to be 5 mg of pilocarpine three times a day, striking a balance between clinical benefits and side effects [63]. However, Nakamura et al. (2009) found that this identical dosage led to a high incidence of unbearable side effects, with sweating being the most frequently reported, affecting 64% of participants [64].

In comparisons between pilocarpine and another sialogogue, cevimeline, there were higher rates of dissatisfaction among first-time pilocarpine users (47% vs. 27%). Excessive sweating was the most common side effect prompting discontinuation of treatment, and it occurred more frequently in patients on pilocarpine (25%) than those using cevimeline (11%) [65]. This observation is consistent with a study by Chainani-Wu et al., where sweating was reported more commonly with pilocarpine than with bethanechol or cevimeline [66].

Pilocarpine has the potential to interact with other drugs, including beta-adrenergic blockers and other parasympathomimetic medications, potentially countering the therapeutic anticholinergic impacts of medicines such as oxybutynin. Therefore, while the systemic administration of pilocarpine offers notable advantages in managing xerostomia, its usage necessitates careful deliberation due to the likelihood of unwanted side effects and interactions with concurrent medications.

##### Topical Administration of Pilocarpine

Various pilocarpine delivery systems, including mouthwashes, tablets, lozenges, and mouth sprays, have been investigated in numerous studies. The administration of pilocarpine topically has shown a comparable efficacy to systemic delivery, while enhancing patient tolerability.

One of the pioneer studies that was a single-blind, placebo-controlled experiment to assess the efficacy of protracted topical application of a pilocarpine solution in patients with Sjögren’s syndrome was carried out by Rhodus et al. (1991). The study identified a substantial increase in both unstimulated whole and stimulated parotid saliva, with minimal adverse effects in the group treated with pilocarpine [67].

Davies et al. (1994) compared mucin-based artificial saliva and a pilocarpine mouthwash in patients with radiation-induced xerostomia. The pilocarpine mouthwash showed increased effectiveness in relieving xerostomia symptoms compared to artificial saliva [68].

Bernardi et al. (2002) conducted a study examining the impacts of pilocarpine mouth rinses on salivary flow and adverse effects in a healthy population, establishing an elevation in salivary flow without any negative outcomes [69]. A similar study by Kim et al. reported that a 0.1% pilocarpine mouth rinse increased the whole salivary flow rate in patients suffering from xerostomia [70].

In 2015, Tanigawa and his colleagues evaluated the effectiveness and safety of pilocarpine mouthwash in elderly patients dealing with xerostomia. The study found a comprehensive improvement in 47% of the individuals in the pilocarpine group compared to a mere 14% in the control group. Mild side effects, primarily oral discomfort, were reported in a minority of patients [71].

In a direct comparison conducted by Park et al. (2015), the impacts of a 2% pilocarpine mouthwash on salivary flow were juxtaposed with the effects of a 5 mg pilocarpine tablet in a population of healthy individuals. The findings suggested that the pilocarpine-infused mouthwash augmented salivary flow rate to a degree akin to the tablet [72].

Song et al.’s study evaluated the effects of mouthwashes with varying pilocarpine concentrations on 30 healthy volunteers divided into six groups. While 0.1% and 0.5% pilocarpine concentrations did not impact salivation, concentrations ≥1% significantly increased salivation without severe side effects [73]. In a similar randomized, placebo-controlled trial by Vera et al. (2018), involving 36 healthy individuals, salivation increase was dose- and time-dependent. Compared to saline control, the 2% pilocarpine solution led to a significant salivation increase 60 and 75 min post administration [74].

Significant enhancements in both stimulated and unstimulated salivary flow rates were noted in patients with residual functioning salivary tissue when they used pilocarpine, even though three patients discontinued early due to adverse events. In a clinical trial conducted by the team of Santos Polvora, which employed a prospective, randomized, double-blind, crossover, and placebo-controlled design, a marked elevation in salivary flow was recorded in participants treated with a pilocarpine spray [75]. However, these findings conflict with Pereira et al.’s study, where the pilocarpine spray’s efficacy was similar to the placebo in 40 patients with radiation-induced xerostomia [76].

In a study by Muthumariappan et al. (2019), pilocarpine-loaded poly(lactic-co-glycolic acid)/polyethylene glycol (PLGA/PEG) nanofiber mats were engineered using an electrospinning process, intending to create a localized formulation that targets the salivary glands. The research demonstrated that this innovative topical application notably augmented saliva secretion, underscoring its potential use as an intradermal formulation for the initial treatment of xerostomia [77].

##### Ginger on Pilocarpine-Stimulated Salivary Flow Rate

Kan et al. (2023) investigated the impact of various extracts of ginger rhizome (GR), specifically 70% (*v*/*v*) methanol, 80% (*v*/*v*) ethanol, and 100% (*v*/*v*) dimethyl sulfoxide (DMSO), as well as 6-shogaol, on pilocarpine-stimulated salivary flow rates in C57BL/6 mice. A total of 30 C57BL/6 mice, all at the age of 15 weeks, were assigned into five distinct experimental subsets: Group 1 (saline control), Group 2 (70% methanol extract), Group 3 (80% ethanol extract), Group 4 (100% DMSO extract), and Group 5 (6-shogaol). Salivary flow rates under pilocarpine stimulation were initially measured at 15 weeks of age, followed by intraperitoneal injection of the treatment solutions during weeks 16 to 18.

Salivary flow rates displayed a statistically significant augmentation (*p* < 0.05) when the treatment groups (Groups 2, 3, 4, and 5) were compared to the initial baseline measurements. All four treatment subsets showed noticeably higher salivary flow rates than the control group, with this difference reaching statistical significance (*p* < 0.05). Group 4 (100% DMSO extract) reported the most pronounced rise in salivary flow rates among the treatment subsets. Nonetheless, the differences in salivary flow rates between the treatment groups were not statistically significant (*p* > 0.05).

Results demonstrated that all ginger rhizome extracts, including 70% methanol, 80% ethanol, 100% DMSO, and 6-shogaol, effectively increased the pilocarpine-stimulated salivary flow rates in C57BL/6 mice [78].

## 4. Summary and Conclusions

Xerostomia, known as dry mouth, is a widespread oral health condition characterized by reduced salivary flow. It can lead to discomfort, difficulty swallowing, and an increased risk of oral infections. This comprehensive review evaluates the therapeutic potential of various natural compounds and extracts for treating xerostomia by examining their effects on salivary flow and the underlying mechanisms involved.

Various natural products exhibit potential pharmacological effects in addressing xerostomia (dry mouth). Studies indicate that specific natural agents may impede salivary dysfunction through molecular pathways, including the suppression of autoantigens, modulation of antioxidant enzymes and influence on cell proliferation. Additionally, the consumption of select natural compounds is linked to an elevation in oral moisture levels, potentially alleviating dry mouth conditions. Furthermore, certain natural products are believed to stimulate the gustatory system, create protective barriers in the oral cavity and possess anti-inflammatory and antioxidant properties that contribute to hydrating mucosal surfaces. Generally, antioxidant supplementation as a potential therapeutic intervention for xerostomia, has attracted considerable interest, particularly in the context of radiotherapy. The rationale lies in the potential protective effect of antioxidants against radiation-induced oxidative stress, a significant contributor to salivary gland damage and subsequent xerostomia. Clinical trials have demonstrated promising results with antioxidant supplementation, underlining its potential as a preventive and therapeutic strategy. However, definitive conclusions about its efficacy and safety are yet to be drawn due to the heterogeneity of these studies in terms of antioxidant types, doses, administration schedules, and outcome measures.

Despite the promising results of these natural compounds and extracts in treating xerostomia, several limitations and knowledge gaps still need to be addressed. Further research is needed to determine the optimal dosages for each compound and the specific targeted molecules, genes, and signaling pathways involved in their therapeutic effects. Moreover, more clinical trials are required to establish the safety and efficacy of these treatments in human populations.

In conclusion, this comprehensive review provides an overview of the current state of knowledge regarding natural compounds and extracts for the treatment of xerostomia. The results demonstrate the potential of these compounds in improving salivary flow and alleviating symptoms associated with xerostomia. While further research is necessary to fully understand the mechanisms underlying the therapeutic effects of these compounds, this review paves the way for developing novel therapeutic strategies for addressing this common oral health issue. With continued investigation, natural compounds and extracts may offer promising alternatives or adjuncts to existing treatments for xerostomia, improving the quality of life for affected individuals.

## Figures and Tables

**Table 1:** In vivo studies evaluating the efficacy of various natural products in relieving the symptoms of xerostomia.

Natural Product/Compound	Product Form	Study on	Outcome/Results	Year of Publication	Reference
Apigenin	Apigenin powder	24 female ICR mice (6-week-old, 30 ± 2 g)	Apigenin treatment increased the salivary secretion index and decreased water consumption in mice. Dose: 50 mg/kg/day, dissolved in 0.5% carboxymethyl cellulose sodium.	2022	[2]
*Ixeris dentata* (Thunb.) Nakai *[Asteraceae]* (IXD)	IXD ethanolic extract	40 Sprague-Dawley male rats	Diabetic rats treated with IXD showed lower saliva and blood glucose concentrations, improved total body weight, and higher expression of salivary α-amylase. Dose: 100 mg/kg body weight ethanolic extract.	2018	[38]
*Ixeris dentata* (Thunb.) Nakai *[Asteraceae]* (IXD)	IXD methanolic suspended in H_2_O	85 Sprague-Dawley adult male rats	In ageing rats, the administration of IXD extract at a dosage of 100 mg/kg led to a significant increase in total saliva compared to younger rats under the same treatment conditions.	2018	[39]
*Lycium barbarum* L. polysaccharide (LBP)	LBP powder (>95% purity)	32 female non-obese diabetic (NOD) mice (7-week-old, 18 ± 2 g)	Low-dose LBP improves the salivary flow rates and alleviates inflammation in submandibular glands (SMGs), positively impacting primary Sjögren’s syndrome disease progression in NOD mice. Dose: 5 and 10 mg kg^−1^ d^−1^	2021	[40]

**Table 2:** Clinical trials evaluating the efficacy of various natural products in relieving the symptoms of xerostomia.

Natural Product/Compound	Product Form	Number of Patients	Outcome/Results	Year of Publication	Reference
*Camellia sinensis* (L.)	*Camellia sinensis* (L.) (green tea) extract lozenge	60	The green tea extract lozenge showed a statistically significant increase in both SWSFR and UWSFR, but no significant difference in the quality of life (QoL).	2014	[41]
*Thymus* L.	Thyme honey	72	Thyme honey was found to be effective in reducing or stabilizing the degree of xerostomia, and also positively affected other symptoms, such as pain, taste loss, dysphagia (difficulty swallowing), QoL, and general satisfaction. Dose: Oral rinses (20 mL of thyme honey diluted in 100 mL of purified water).	2017	[42]
*Vaccinium vitis-idaea* L. (Common name: lingonberry)	Fermented lingonberry juice (FLJ) (Lingora^®^, Vantaa, Finland)	21	FLJ mouthwash positively affected all five salivary parameters (resting and stimulated saliva secretion rates, resting saliva pH, and stimulated saliva buffering capacity), with statistically significant improvements. Dose: 10 mL daily for 30 s.	2022	[43]
*Cocos nucifera* L. (Common name: coconut)	Coconut oil	30	There was no significant difference in xerostomia quality of life scale (XeQoLS) scores pre- and post-treatment among the entire study group and participants who continued coconut oil beyond the study period. Dose: Coating patient’s mouth with coconut oil prior to meals and at bedtime.	2020	[44]
Lycopene enriched *Olea europaea* L. (olive)	Lycopene enriched olive oil spray	60	Patient-assessed benefits and satisfaction with the spray were higher in the treatment group but the difference between treatment and placebo groups was not statistically significant. Dose: 1.5 mL spray to the mouth, three times per day.	2017	[45]
*Zingiber officinale* Roscoe (Common name: ginger)	Ginger ethanolic extract in a form of oral spray	20	The ginger herbal spray effectively increased salivation and reduced the severity of dry mouth in patients with type II diabetes.	2017	[46]
*Zingiber officinale* Roscoe (Common name: ginger)	Ginger capsule (Zintoma, Goldaru Company, Iran)	61	Marginally significant improvement in xerostomia symptoms with ginger treatment. Still, there were no significant differences between the groups regarding improvement of dry mouth symptoms and quality of life issues, except for dry mouth experience throughout the day. Dose: three capsules daily.	2017	[47]
*Zingiber officinale* Roscoe (Common name: ginger)	Ginger mouthwash (25% ginger)	105	Ginger mouthwashes significantly reduced all xerostomia symptoms (need to drink water to swallow dry foods, decreased salivation, mouth dryness upon waking, dry mouth during travel, and burning mouth sensation. Dose: 20 mL three times daily for 14 consecutive days.	2021	[48]
*Aloe vera* (L.) Burm.f. (Common name: Aloe vera)	Aloe vera mouthwash (50% aloe vera)	105	Aloe vera mouthwashes significantly reduced all xerostomia symptoms (need to drink water to swallow dry foods, decreased salivation, mouth dryness upon waking, dry mouth during travel, and burning mouth sensation. Dose: 20 mL three times daily for 14 consecutive days.	2021	[48]
*Aloe vera* (L.) Burm.f. and *Mentha* L. (Peppermint)	Moisturizing gel (Veramin)	80	The results indicated that the gel (Veramin) had significant effects on reducing mouth dryness and improving oral health. Dose: Apply gel to all surfaces of the oral mucosa, the gum, and the tongue after brushing, every 4 h.	2018	[49]
Glucosylceramide	Tablets containing Glucosylceramide Extracted from *Ananas comosus* (L.) Merr. (Common name: Pineapple) (GCP)	16	GCP administration significantly increased the oral moisture level of the lingual mucosa and the visual analog scale value related to xerostomia. Dose: one tablet per day (1.2 mg GCP per tablet).	2019	[50]
*Linum usitatissimum* L. (also known as linseed)	Linseed extracts: Salinum by Camurus AB, Sweden and MAS-84	20	Both tested preparations had a beneficial effect on dry mouth symptoms, with Salinum showing improved results and performance. Dose: 2 mL.	1995	[51]
*Matricaria chamomilla* L. (Common name: Chamomile) and *Linum usitatissimum* L. (also known as linseed)	Chamomile and linseed saliva substitute	74	The chamomile and linseed saliva substitute positively affected four out of five xerostomia symptoms, while the conventional saliva substitute only impacted two of them. Dose: 2 mL, four times per day.	2017	[52]
*Hibiscus sabdariffa*	Aqualief™ (Helsinn Healthcare SA, Lugano, Switzerland) tablets	60	Aqualief™ significantly improved dry mouth symptoms compared to the placebo (normalizing saliva pH and significantly increase the saliva flow rate). Dose: Three times/day (after meals) for six consecutive days.	2020	[53]
*Malva sylvestris* L. and *Alcea digitata* (Boiss.)	*Malva sylvestris* and *Alcea digitata* in powdered form	62	The experimental and control groups revealed a significant decrease in the visual analog scale (VAS) score for the experimental group at four weeks when compared to the control group. Dose: Three times per day for four weeks.	2016	[54]
Vitamin C/E Complex	Antioxidant supplements	45	Short-term supplementation with an antioxidant vitamin E/C complex protects against RT-induced xerostomia in patients with head and neck cancer. Dose: Twice per day.	2016	[55]
*Plantago ovata* Forssk.	*Plantago ovata* husk in water as mouthwash	28	The herbal compound significantly mitigated oral mucositis, pain, xerostomia, and improved life quality versus placebo (*p* < 0.05); the oral care protocol likewise reduced oral mucositis. Dose: Three times per day.	2020	[56]
Pilocarpine	Oral tablets (systemic administration)		Systemic administration of pilocarpine increases resting and stimulated salivary flow, decreases subjective oral dryness, and improves chewing and speaking abilities of patients. Dose: Ranging from 2.5 to 10 mg, taken orally three or four times daily	2022	[12]

## Data Availability

Not applicable.
